# *TP53* mutations in *de novo* acute myeloid leukemia patients: longitudinal follow-ups show the mutation is stable during disease evolution

**DOI:** 10.1038/bcj.2015.59

**Published:** 2015-07-31

**Authors:** H-A Hou, W-C Chou, Y-Y Kuo, C-Y Liu, L-I Lin, M-H Tseng, Y-C Chiang, M-C Liu, C-W Liu, J-L Tang, M Yao, C-C Li, S-Y Huang, B-S Ko, S-C Hsu, C-Y Chen, C-T Lin, S-J Wu, W Tsay, Y-C Chen, H-F Tien

**Affiliations:** 1 Division of Hematology, Department of Internal Medicine, National Taiwan University Hospital, Taipei, Taiwan; 2 Department of Laboratory Medicine, National Taiwan University Hospital, Taipei, Taiwan; 3 Graduate Institute of Oncology, College of Medicine, National Taiwan University, Taipei, Taiwan; 4 Biostatistics Consulting Laboratory, Department of Nursing, National Taipei College of Nursing, Taipei, Taiwan; 5 Department of Clinical Laboratory Sciences and Medical Biotechnology, College of Medicine, National Taiwan University, Taipei, Taiwan; 6 Department of Pathology, National Taiwan University Hospital, Taipei, Taiwan; 7 Tai-Chang Stem Cell Therapy Center, National Taiwan University, Taipei, Taiwan

## Abstract

The *TP53* mutation is frequently detected in acute myeloid leukemia (AML) patients with complex karyotype (CK), but the stability of this mutation during the clinical course remains unclear. In this study, *TP53* mutations were identified in 7% of 500 patients with *de novo* AML and 58.8% of patients with CK. *TP53* mutations were closely associated with older age, lower white blood cell (WBC) and platelet counts, FAB M6 subtype, unfavorable-risk cytogenetics and CK, but negatively associated with *NPM1* mutation, *FLT3/*ITD and *DNMT3A* mutation. Multivariate analysis demonstrated that *TP53* mutation was an independent poor prognostic factor for overall survival and disease-free survival among the total cohort and the subgroup of patients with CK. A scoring system incorporating *TP53* mutation and nine other prognostic factors, including age, WBC counts, cytogenetics and gene mutations, into survival analysis proved to be very useful to stratify AML patients. Sequential study of 420 samples showed that *TP53* mutations were stable during AML evolution, whereas the mutation was acquired only in 1 of the 126 *TP53* wild-type patients when therapy-related AML originated from different clone emerged. In conclusion, *TP53* mutations are associated with distinct clinic-biological features and poor prognosis in *de novo* AML patients and are rather stable during disease progression.

## Introduction

Somatic mutation of the tumor suppressor gene, *TP53*, located in 17p13 is one of the most frequent alterations in cancer.^1, 2^ The TP53 protein exerts its tight regulation of apoptosis and cell cycle integrity, and inactivation of TP53 may lead to uncontrolled cell proliferation and promote cancer development.^3, 4^ The frequency of *TP53* mutation is usually increased in the patients with advanced stages or aggressive types of cancers.^5, 6^

Several studies have shown *TP53* mutations are frequently detected in patients with therapy-related acute myeloid leukemia (AML)^7^ or AML with complex karyotype (AML-CK).^8, 9, 10^ The incidences of this mutation in AML-CK varied from 53% in a British series,^10^ to 60–69% in two German studies.^8, 9^ In contrast, *TP53* mutations rarely occurred in patients without CK (2.1%)^8^ or 17p chromosomal abnormality (2.8%).^11^ The reports regarding the prognostic relevance of *TP53* mutations in patients with AML-CK showed controversial results. Rucker *et al.*^9^ showed that *TP53* mutation was an independent poor risk factor for overall survival (OS) in AML patients with CK; however, the same finding could not be shown by Bowen *et al.*^10^

Whether there is geographic difference in the incidence of *TP53* mutations in AML between Western and Asian people remains to be determined. In addition, the interaction of *TP53* mutations with other genetic alterations in AML was largely unknown. Furthermore, to the best of our knowledge, there has been no report in literature concerning the stability of *TP53* mutations during the clinical course. In this study, we investigated *TP53* mutation in 500 patients with *de novo* AML and analyzed its interactions with 17 other genetic alterations. Longitudinal follow-ups of the status of *TP53* mutation during the clinical course were also performed in 131 patients to investigate the stability and pathogenic role of this mutation in AML. Furthermore, to better stratify AML patients into different risk groups, a scoring system integrating *TP53* mutations with nine other prognostic factors, including age, white blood cell (WBC) count, cytogenetics, *NPM1/FLT3-*ITD, *CEBPA*, *RUNX1*, *WT1*, *DNMT3A* and *IDH2* mutations, into survival analysis was proposed.

## Materials and methods

### Subjects

From March 1995 to December 2008, a total of 500 adult patients who were newly diagnosed as having *de novo* AML at the National Taiwan University Hospital and had enough cryopreserved cells for analysis were enrolled consecutively. Patients with antecedent hematological diseases or therapy-related AML were excluded. Diagnosis and classification of AML were made according to the FAB (French–American–British) Cooperative Group Criteria. Among them, 363 (72.6%) patients received standard induction chemotherapy (Idarubicin 12 mg/m^2^ per day on days 1–3 and Cytarabine 100 mg/m^2^ per day on days 1–7) and then consolidation chemotherapy with 2–4 courses of high-dose Cytarabine (2000 mg/m^2^ q.12 h, days 1–4, total 8 doses), with or without an anthracycline (Idarubicin or Novatrone), after achieving complete remission (CR).^12, 13^ The patients with acute promyelocytic leukemia (M3 subtype) received concurrent all-*trans* retinoic acid and chemotherapy. The remaining 137 patients received palliative therapy with supportive care and/or low-dose chemotherapy because of underlying comorbidity or based on the decision of the patients. A total of 45 patients received allogeneic hematopoietic stem cell transplantation in first CR. This study was approved by the institutional review board of the National Taiwan University Hospital; and written informed consent was obtained from all participants in accordance with the Declaration of Helsinki.

### Cytogenetics

Bone marrow (BM) cells were harvested directly or after 1–3 days of unstimulated culture as described previously.^14^ Metaphase chromosomes were banded by trypsin-Giemsa technique and karyotyped according to the International System for Human Cytogenetic Nomenclature.

### Immunophenotype analysis

A panel of monoclonal antibodies to myeloid-associated antigens, including CD13, CD33, CD11b, CD15, CD14 and CD41a, as well as lymphoid-associated antigens, including CD2, CD5, CD7, CD19, CD10 and CD20, and lineage nonspecific antigens HLA-DR, CD34 and CD56 were used to characterize the phenotypes of the leukemia cells as previously described.^12^

### Mutation analysis

Mutation analysis of *TP53* exons 3–9 was performed by PCR and direct sequencing according to previous reports with mild modification.^8, 9^ The primer sequences are shown in Supplementary Table 1. Abnormal sequencing results were confirmed by at least two repeated analyses. Sequential analysis of *TP53* mutation during the clinical course was performed in 420 samples from 131 patients. Mutation analyses of 17 other relevant molecular marker genes, including class I mutations such as *FLT3/*ITD and *FLT3/*TKD,^15^ *NRAS*,^16^ *KRAS*,^16^ *JAK2*,^16^ *KIT*^17^ and *PTPN11* mutations,^18^ and class II mutations such as *CEBPA*^19^ and *RUNX1* mutations,^20^ as well as *NPM1*,^21^ *WT1*,^22^ and those genes related to epigenetic modification such as *MLL/*PTD,^23^ *ASXL1*,^24^ *IDH1*,^25^ *IDH2*,^26^ *TET2*,^27^ and *DNMT3A* mutations^12^ were performed as previously described. To detect *TP53* mutation at diagnosis, we used DNA amplified *in vitro* from patients' BM cells by Illustra GenomiPhi V2 DNA amplification kit as described by the manufacturer (GE Healthcare, Buckinghamshire, UK). All the mutations detected in such samples were verified in the original nonamplified samples.

### TA cloning analysis

For the patients with discrepancy of the mutation status of the *TP53* in paired samples, Taq polymerase-amplified (TA) cloning was performed in the samples without detectable mutant by direct sequencing. The DNA spanning the mutation spots of *TP53* detected at either diagnosis or during subsequent follow-ups was amplified and the PCR products were then cloned into the TA cloning vector pGEM-T Easy (Promega, Madison, WI, USA). Direct sequencing was then performed on the selected clones. More than 40 clones were selected for sequencing as previously described.^28^

### Statistical analysis

The discrete variables of patients with and without *TP53* mutation were compared using the χ^2^ tests, but if the expected values of contingency tables were <5, Fisher's exact test was used. If the continuous data were not normally distributed, Mann–Whitney *U*-tests were used to compare continuous variables and medians of distributions. To evaluate the impact of *TP53* mutation on clinical outcome, only the patients who received conventional standard chemotherapy, as mentioned above, were included in the analysis.^12, 13^ OS was measured from the date of first diagnosis to the date of last follow-up or death from any cause, whereas relapse was defined as a reappearance of at least 5% leukemic blasts in a BM aspirate or new extramedullary leukemia in patients with a previously documented CR.^29^ Disease-free status indicated that the patient achieved CR and did not relapse by the end of this study. Cox regression survival estimation was used to plot survival curves and to test the difference between groups. Multivariate Cox proportional hazard regression analysis was used to investigate independent prognostic factors for OS and disease-free survival (DFS). The proportional hazards assumption (constant hazards assumption) was examined by using time-dependent covariate Cox regression before conducting multivariate Cox proportional hazard regression. The variables including age, WBC counts, karyotype, *NPM1/FLT3-*ITD, *CEBPA*, *IDH2*, *WT1*, *RUNX1*, *ASXL1*, *DNMT3A* and *TP53* mutations were used as covariates. Those patients who received hematopoietic stem cell transplantation were censored at the time of hematopoietic stem cell transplantation in survival analysis to ameliorate the influence of the treatment.^12, 13^ A *P*-value of <0.05 was considered statistically significant. All statistical analyses were performed with the SPSS 19 (SPSS Inc., Chicago, IL, USA) and Statsdirect (Cheshire, UK).

## Results

### *TP53* mutations in patients with *de novo* AML

**Figure 1:**
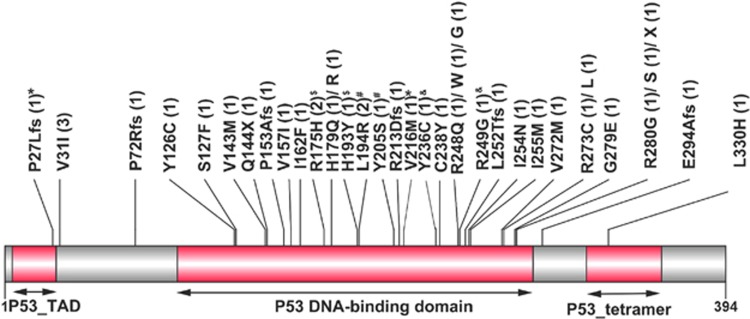
Patterns and locations of the *TP53* mutations. The positions and predicted translational consequences of *TP53* mutations detected in 500 AML samples are shown. The number of patients with the mutation is indicated in the parenthesis behind each mutation. The symbols ‘#', ‘*', ‘&', and ‘$' indicate that patients have two mutations.

A total of 36 different *TP53* mutations were identified in 35 patients (Table 1 and Figure 1). Of these, 28 were missense mutations, 2 were nonsense mutations, 5 were frame-shift mutations and 1 was in-frame mutation. V31I occurred in three patients, R175H and L194R in two each and all other mutations in only one each. Five patients had double heterozygous mutations (patients 1, 4, 14, 21 and 23). The remaining 30 patients showed only one mutation; all were heterozygous.

### Correlation of *TP53* mutations with clinical and laboratory features

In total, 500 *de novo* AML patients, including 35 (7%) *TP53*-mutated and 465 *TP53* wild-type patients, were enrolled into the analysis. The comparison of clinical characteristics of patients with and without *TP53* mutations is shown in Table 2.

*TP53*-mutated patients were older (median, 67 vs 50 years, *P*=0.0003) and had lower WBC, blast and platelet counts than *TP53* wild-type patients (*P*<0.0001, <0.0001 and 0.0267, respectively). Patients with FAB M6 subtype of AML had the highest incidence of *TP53* mutation than those with other subtypes. The mutations were positively associated with the expression of CD34 on the leukemic cells (Supplementary Table 2). There was no difference in the expression of other antigens between the patients with and without *TP53* mutation.

### Association of *TP53* mutations with cytogenetic abnormalities

Chromosome data were available in 482 patients at diagnosis, including 35 *TP53-*mutated and 447 *TP53* wild-type patients (Supplementary Table 3). *TP53* mutations occurred more frequently in patients with unfavorable-risk cytogenetics (46.2%) than in those with favorable- or intermediate-risk cytogenetics (1.2%, *P*<0.0001). There was also a significant difference in the incidence of the *TP53* mutation among patients with normal karyotype (1.8%), simple chromosomal abnormalities with one or two changes (0.5%) and CK with three or more abnormalities (58.8%, *P*<0.0001). Besides, *TP53-*mutated patients had a higher degree of karyotypic complexity, as defined by five or more chromosomal changes, than *TP53* wild-type patients in the subgroup of patients with CK (90% vs 42.9%, *P*=0.0005). None of the patients with t(15;17), inv(16), t(7;11) or 11q23 translocations showed *TP53* mutation, but one patient with t(8;21) harbored this mutation concurrently. There was no association of *TP53* mutation with other chromosomal abnormalities, including +8, +11, +13, +21, −5/del(5q) and −7/del(7q).

### Association of *TP53* mutation with other molecular abnormalities

To investigate the interaction of gene mutations in the pathogenesis of adult AML, a complete mutational screening of 17 other genes was performed in all 500 patients (Table 3). Among the 35 patients with *TP53* mutations, 13 (37.1%) showed additional molecular abnormalities at diagnosis (Tables 1 and 4). Nine had one additional change and four had two. The associated molecular events included *NRAS*, *PTPN11*, *CEBPA*, *RUNX1*, *ASXL1* and *TET2* mutations that each occurred in two patients. Patients with *TP53* mutations had significantly lower incidences of *NPM1* mutation, *FLT3/*ITD and *DNMT3A* mutations than those with *TP53* wild-type (2.9% vs 21.9%, *P*=0.0041; 0% vs 24.3%, *P*=0.0002; and 2.9% vs 14.8% *P*=0.045, respectively). There was no difference in the incidence of other molecular mutations between patients with and without *TP53* mutation. Interestingly, *TP53*-mutated patients with complex cytogenetics had lower probability of concurrent other molecular alterations than those without (26.7% vs 100%, *P*=0.004).

### Impact of *TP53* mutation on response to therapy and clinical outcome

**Figure 2:**
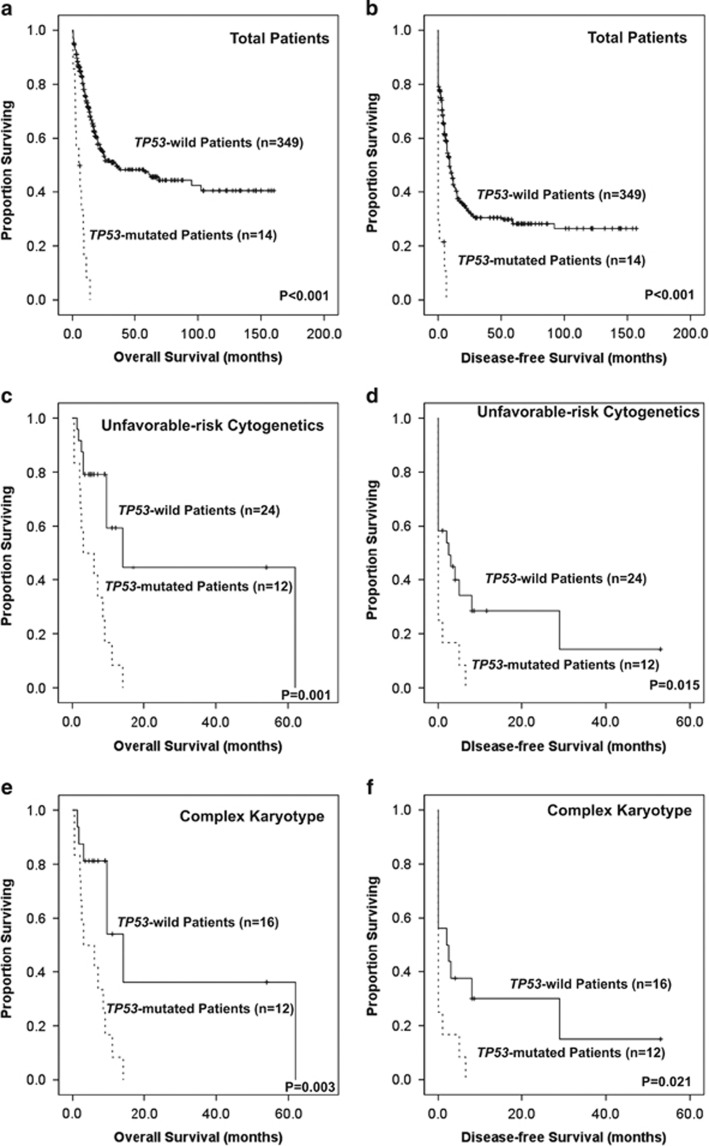
Kaplan–Meier survival curves for OS and DFS in a total of 363 AML patients (**a**, **b**), 36 patients with unfavorable-risk cytogenetics (**c**, **d**) and 28 patients with complex karyotype (**e**, **f**) who received standard intensive chemotherapy.

Of the 363 AML patients undergoing conventional intensive induction chemotherapy, 284 (78.5%) patients achieved a CR. *TP53* mutation was associated with an inferior response rate (CR rate 28.6% vs 80.2%, *P*<0.0001) and higher probability to be refractory to treatment (50% vs 13.5%, *P*=0.0017). With a median follow-up of 55 months (range, 1.0–160), patients with *TP53* mutation had significantly poorer OS and DFS than those without *TP53* mutation (median, 5 vs 35 months, *P*<0.001, and median, 0 vs 9 months, *P*<0.001, respectively, Figures 2a and b). In the subgroups of 36 patients with unfavorable-risk cytogenetics, the differences in OS and DFS were still significant between patients with and without *TP53* mutation (median, 9.5 vs 14 months, *P*=0.001, Figure 2c and median, 0 vs 2.5 months, *P*= 0.015, Figure 2d, respectively). The same was also true for the subgroup of 28 patients with CK (median, 9 vs 14 months, *P*=0.003, Figure 2e and median, 0 vs 2 months, *P*=0.021, Figure 2f, respectively). Intriguingly, among the patients without *TP53* mutation, the OS is similar between those with intermediate-risk cytogenetics and unfavorable-risk cytogenetics (*P*=0.304).

In multivariate analysis (Table 4), the independent poor risk factors for OS were older age >50 years, high WBC counts >50 000/μl, unfavorable-risk cytogenetics and *TP53*, *RUNX1*, *WT1* and *DNMT3A* mutations. On the other hand, *CEBPA*^double mutation^ and *NPM1* mutation in the absence of *FLT3-*ITD (*NPM1*^+^/*FLT3-*ITD^-^) were independent favorable prognostic factors. There was a trend of better OS in patients with *IDH2* mutation (hazard ratio 0.563, 95% confidence interval 0.292–1.086, *P*=0.087). Similarly, the independent poor risk factors for DFS included older age >50 years, high WBC counts >50 000/μl, unfavorable-risk cytogenetics and *TP53*, *RUNX1*, *WT1* and *DNMT3A* mutations. On the other hand, *CEBPA*^double mutation^ and *NPM1*^+^/*FLT3-*ITD^-^ were independent favorable prognostic factors.

**Figure 3:**
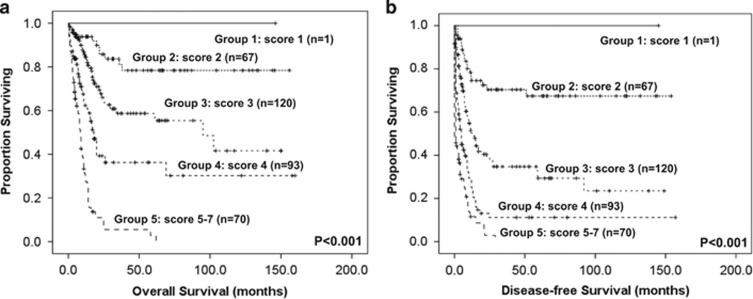
Kaplan–Meier survival curves for OS (**a**) and DFS (**b**) in AML patients based on scoring system (*P*<0.001 for both OS and DFS). AML patients were grouped according to scoring system based on *TP53* mutation and 9 other prognostic markers (*CEBPA*^double-mutation^, *NPM1*/*FLT3-*ITD, *IDH2*, *TP53, WT1*, *RUNX1* and *DNMT3A* mutations, age and WBC counts at diagnosis). A score of −1 was assigned for each parameter associated with a favorable outcome (*CEBPA*^double mutation^, *IDH2* mutation and *NPM1*^+^/*FLT3-*ITD^-^), whereas a score of +1 was assigned for each factor associated with an adverse outcome (*TP53*, *WT1*, *RUNX1* and *DNMT3A* mutations, older age and higher WBC counts at diagnosis). The karyotypes were stratified into three groups (unfavorable: +2, intermediate: +1 and favorable: 0). The algebraic summation of these scores of each patient was the final score. The 12 patients without chromosome data were not included in the analysis.

To better stratify the AML patients into different risk groups, a scoring system incorporating 10 prognostic markers, including age, WBC counts, cytogenetics at diagnosis, *NPM1*/*FLT3-*ITD and mutations of *CEBPA*, *IDH2, TP53, DNMT3A*, *RUNX1* and *WT1*, into survival analysis was formulated based on the results of our Cox proportional hazards model. A score of −1 was assigned for each parameter associated with a favorable outcome (*CEBPA*^double mutation^, *IDH2* mutation and *NPM1*^+^/*FLT3-*ITD^-^), whereas a score of +1 for each factor associated with an adverse outcome (*TP53, DNMT3A*, *WT1* and *RUNX1* mutations, older age and higher WBC counts at diagnosis). The karyotypes were stratified into three groups (unfavorable: +2, intermediate: +1 and favorable: 0). The algebraic summation of these scores of each patient was the final score. This score system divided the AML patients into five groups with different clinical outcomes (*P*<0.001 for both OS and DFS, Figure 3).

### Sequential studies of *TP53* mutations

*TP53* mutations were serially studied in 420 samples from 131 patients, including 5 patients with *TP53* mutations and 126 patients without this mutation at diagnosis (Table 5). Among the five patients with *TP53* mutations who had ever obtained a CR and had available samples for study, three lost the original mutation at remission status, but two (patients 11 and 32) retained it (Table 5). These two patients relapsed soon and died of uncontrolled disease. In the three patients who had available samples for serial study at relapse, the original mutation could be detected at relapse in two patients (patients 11 and 12), but was lost in one (patient 30). Because direct sequencing might not be sensitive enough to detect low level of *TP53* mutation signal, we therefore sequenced TA clones of the PCR product from patient 30 at relapse. Two mutant clones out of 45 were detected. Among the 126 patients who had no *TP53* mutation at diagnosis, one (patient 36) acquired *TP53* mutation at second relapse. Patient 36 was diagnosed as having acute promyelocytic leukemia with karyotypic change of t(15;17) and trisomy 8 (Table 5). He acquired *TP53* mutation at second relapse, 106 months after the initial study at diagnosis and 39.5 months after CR2. At that time, karyotypic evolution with complex chromosomal change but loss of the original t(15;17) and trisomy 8 was found. Because cytogenetic analysis might not be sensitive to detect minor clones, we performed both fluorescence *in situ* hybridization and real-time quantitative PCR for the *PML-RARA* fusion transcript. No *PML-RARA* mutant was detected at second relapse. Intriguingly, we could identify *TP53* mutation in 1 of 39 clones by a sensitive cloning technique at first relapse when t(15;17) was still present. However, we did not find *TP53* mutant in the BM cells from the patients at diagnosis even after using a sensitive technique.

## Discussion

In this study, we found that the *TP53* mutation was associated with distinct clinic-biological features and was a poor prognostic factor in AML patients, independent of age, WBC counts, karyotype and other genetic markers.

A total of 36 *TP53* mutations, most commonly in the DNA-binding domain, were detected in 7% of patients (Figure 1). The majority were missense mutations that were suggested to abolish the DNA-binding activity and transactivation capacity.^3^ Overall, three involved exon 4, nine exon 5, six exon 6, nine exon 7, nine exon 8 and none involved exon 9. Most *TP53* mutations were found in exons 5–8,^30, 31^ and few mutations occurred outside exons 5–8.^9^ We analyzed exons 3–9 in this study to avoid missing some mutations outside exons 5–8. The probability that the *TP53* wild-type patients in this study would have mutations outside the area we screened was low, though it could not be totally excluded.

Most studies on *TP53* mutations were focused on patients with CK, 17p abnormalities or older population.^9, 32, 33^ In this study, we analyzed 500 consecutive patients, both cytogenetically normal and abnormal, so the frequency and clinical characteristics of *TP53* mutations in unselected *de novo* AML patients could be known. *TP53* mutations were found in 7%, 1%, 1.3% and 46.2%, respectively, in whole cohort and in patients with favorable-, intermediate- and unfavorable-risk cytogenetics. The patients with CK had the highest incidence (58.8%) of *TP53* mutations, an incidence similar to two previous reports (53–60%),^9, 10^ but lower than that of Haferlach *et al.*^8^ (69% in 149 patients). The reason of the variability in the incidence of *TP53* mutations in different studies is unknown but may be because of the differences in ethnic background, patient populations recruited and methods used. We also found that *TP53-*mutated patients had a higher degree of karyotypic complexity than *TP53* wild-type patients in the subgroup of patients with CK (90% vs 42.9%, *P*=0.0005).

Although a close association was observed between *TP53* mutations and a complex karyotype, little is known about the interaction between *TP53* mutations and other molecular genetic alterations in AML patients. In a study of mutational status of *TP53*, *NPM1*, *MLL*/PTD and *FLT3* in 235 patients, including 214 with *de novo* AML, 13 therapy-related AML and 8 AML evolving from myelodysplastic syndrome, 1 of the 33 *TP53*-mutated patients had concurrent *MLL*/PTD and another 1 patient had *FLT3* length mutation.^8^ *NPM1* mutation was not observed in patients with *TP53* mutation. Similarly, only 2 patients had concurrent *TP53* and *FLT3* or *RAS* mutations in 140 elderly patients studied.^32^ In the comprehensive analyses of the 17 gene mutations in 500 patients, we found that 13 (37.1%) of 35 patients with *TP53* mutations showed additional molecular abnormalities at diagnosis, including *NRAS*, *PTPN11*, *CEBPA*, *RUNX1*, *ASXL1* and *TET2* mutations that occurred in 2 patients each. Patients with *TP53* mutations had significantly lower incidences of *NPM1* mutation, *FLT3/*ITD and *DNMT3A* mutations than those with *TP53* wild-type. Interestingly, *TP53*-mutated patients with CK had lower probability of concurrent other molecular alterations than those without.

To the best of our knowledge, this study recruited the largest number of AML patients for sequential analysis of *TP53* mutations during the clinical course. In contrast to the instability of *FLT3*-ITD during disease evolution,^34^ we found that the *TP53* mutation seemed rather stable, analogous to *DNMT3A* mutations.^12^ At relapse, the original *TP53* mutations in all three *TP53*-mutated patients studied were retained, but the mutant level in one of them was much reduced at the time of AML relapse, as it could only be detected by a sensitive cloning technique but not by direct sequencing (patient 30, Table 5). On the other hand, among the 126 patients who had no *TP53* mutation at diagnosis, 1 acquired a novel *TP53* mutation at second relapse. This patient was diagnosed as having acute promyelocytic leukemia with t(15;17). She acquired *TP53* mutation at second relapse, 106 months after the initial study at diagnosis; the leukemic cells showed complex chromosomal changes with loss of the original t(15;17) and trisomy 8 at that time (Table 5). It is most likely that the patient developed therapy-related leukemia. We did not find *TP53* mutant in the BM cells from the patients at diagnosis even after using a sensitive cloning technique; intriguingly, we could identify *TP53* mutation in 1 of 39 clones at first relapse of the original leukemia 65 months after diagnosis when t(15;17) was still present. In other words, the minor clone with *TP53* mutant already emerged 41 months before the development of therapy-related leukemia; the minor clone of cells escaped subsequent treatments, expanded and finally transformed to AML accompanied by complex cytogenetic abnormalities.^35^ Taken together, *TP53* mutations are quite stable during AML progression. The acquisition of novel *TP53* mutations in *TP53* wild-type patients may be an indicator of the emergence of therapy-related AML and warrants intervention treatment.

*TP53* mutations within the DNA-binding domain have been associated with poor treatment response and shorter survival in solid tumors.^3^ Regarding the prognostic relevance of *TP53* mutations in AML-CK, Rucker *et al.*^9^ showed that *TP53* mutation was the most important prognostic factor, outweighing all other variables, but another study demonstrated that there was no significant difference in CR and OS between *TP53-*mutant and *TP53* wild-type patients in this group.^10^ In this study, we distinctly identified that patients with *TP53* mutations had poor prognosis in both total cohort and AML-CK. To better stratify AML patients into different risk groups, a survival scoring system incorporating *TP53* mutation and nine other prognostic factors, including age, WBC counts, cytogenetics, *NPM1/FLT3-*ITD, *CEBPA*, *IDH2, RUNX1*, *WT1* and *DNMT3A* mutations, into survival analysis was formulated. Indeed, this scoring system was more powerful than single marker to separate patients into different prognostic groups. Further studies in independent cohorts are needed to validate the clinical implication of the proposed scoring system.

In summary, this study demonstrated that *TP53*-mutated patients had specific clinic-biologic features and cytogenetic changes. *TP53* mutations were mutually exclusive with *NPM1* mutation, *FLT3/*ITD and *DNMT3A* mutations. Furthermore, the *TP53* mutation was an independent poor-risk factor for OS and DFS among total cohort and AML-CK patients. Incorporation of *TP53* mutation with nine other prognostic factors into survival analyses can better stratify AML patients into different risk groups. Sequential study during the clinical course showed that *TP53* mutation was quite stable during AML evolution. The acquisition of *TP53* mutation in *TP53* wild-type patients during clinical follow-ups may be an indicator of the emergence of therapy-related leukemia.

## Figures and Tables

**Table 1:** The mutation patterns in 35 patients with *TP53* mutations at diagnosis

*UPN*	*Age/sex*	*FAB*	*TP53 mutation*			*Other accompanied gene mutations*
			*Location*	*DNA change*	*Protein change*	
1	60/M	M1	Exon 6	c.581T>G	L194R	*PTPN11, RUNX1*
			Exon 6	c.614A>C	Y205S	
2	70/M	M4	Exon 4	c.91G>A	V31I	*IDH1*
3	60/M	M2	Exon 6	c.636del	R213DfsX34	*─*
4	58/F	M8	Exon 4	c.80delC	P27LfsX17	*─*
			Exon 6	c.646G>A	V216M	
5	78/M	M1	Exon 8	c.814G>A	V272M	*─*
6	41/F	M1	Exon 7	c.743G>A	R248Q	*─*
7	79/F	M2	Exon 7	c.752_754del	L252TfsX142	*─*
8	47/F	M6	Exon 8	c.989T>A	L330H	*─*
9	66/F	M2	Exon 8	c.817C>T	R273C	*─*
10	67/M	M0	Exon 8	c.840A>T	R280S	*ASXL1*
11	51/F	M6	Exon 6	c.581T>G	L194R	*─*
12	37/F	M1	Exon 7	c.742C>G	R248G	*─*
13	67/M	M2	Exon 7	c.761T>A	I254N	*NRAS*
14	43/F	M8	Exon 7	c.707A>G	Y236C	*WT1*
			Exon 7	c.745A>G	R249G	
15	66/F	M4	Exon 8	c.836G>A	G279E	*TET2*
16	58/M	M1	Exon 5	c.430C>T	Q144X	*─*
17	72/M	M4	Exon 5	c.484A>T	I162F	*CEBPA, TET2*
18	81/F	M1	Exon 7	c.742C>T	R248W	*─*
19	36/F	M2	Exon 5	c.469G>A	V157I	*ASXL1*
20	74/F	M4	Exon 5	c.450_451insC	P153AfsX28	*NRAS*
21	72/M	M2	Exon 6	c.524G>A	R175H	*─*
			Exon 6	c.577C>T	H193Y	
22	37/F	M6	Exon 7	c.713G>A	C238Y	*─*
23	72/M	M2	Exon 5	c.536A>G	H179R	*─*
			Exon 8	c.838A>T	R280X	
24	68/F	M2	Exon 8	c.879_880del	E294AfsX11	*─*
25	74/M	M8	Exon 4	c.215_225del	P72RfsX73	*─*
26	54/M	M2	Exon 5	c.524G>A	R175H	*─*
27	43/F	M1	Exon 5	c.427G>A	V143M	*─*
28	83/M	M2	Exon 5	c.380C>T	S127F	*─*
29	80/M	M1	Exon 8	c.818G>T	R273L	*RUNX1*
30	71/F	M1	Exon 5	c.377A>G	Y126C	*─*
31	79/F	M4	Exon 7	c.764T>A	I255N	*DNMT3A*
32	30/F	M1	Exon 4	c.91G>A	V31I	*CEBPA, IDH2*
33	66/F	M1	Exon 8	c.838A>G	R280G	*─*
34	72/M	M2	Exon 4	c.91G>A	V31I	*NPM1, PTPN11*
35	66/F	M2	Exon 5	c.537T>G	H179Q	*─*

Abbreviations: F, female; FAB, French–American–British; M, male; UPN, unique patient number.

**Table 2:** Comparison of clinical and laboratory features between AML patients with and without *TP53* mutation

*Variables*	*Total (*n*=500)*	*TP53 mutated (*n*=35, 7%)*	*TP53 wild-type (*n*=465, 93%)*	P-*value*
*Sex*^a^				0.3763
Male	285	17 (6)	268 (94)	
Female	215	18 (8.4)	197 (91.6)	
Age (years)^b^	51 (15–90)	67 (30–83)	50 (15–90)	0.0003

*Lab data*^b^				
WBC (/μl)	19 075 (120–627 800)	3690 (720–178 400)	22 510 (120–627 800)	<0.0001
Hb (g/dl)	8 (2.9–16.2)	7.4 (4.5–12.7)	8 (2.9–16.2)	0.1772
Platelet ( × 1000 /μl)	42 (2–802)	24 (3–802)	44 (2–712)	0.0267
Blast (/μl)	7401 (0–456 725)	1145 (0–100 974)	9744 (0–456 725)	<0.0001
LDH (U/l)	889 (206–15 000)	751 (274–15 000)	860 (206–13 130)	0.3508

*FAB*^a^				
M0	10	1 (10)	9 (90)	0.5193
M1	112	11 (9.8)	101 (90.2)	0.2067
M2	171	12 (7)	159 (93)	>0.9999
M3	38	0 (0)	38 (100)	0.0976
M4	124	5 (4)	119 (96)	0.1584
M5	24	0 (0)	24 (100)	0.3994
M6	12	3 (25)	9 (75)	0.0447
Undetermined	9	3 (33.3)	6 (66.7)	0.0198
*Induction response*^c^	363	14	349	
CR	284	4 (28.6)	280 (80.2)	<0.0001
PR/refractory	54	7 (50)	47 (13.5)	0.0017
Induction death	25	3 (21.4)	22 (6.3)	0.0634
Relapse^c^	144	3 (75)	141 (50.4)	0.6225

Abbreviations: AML, acute myeloid leukemia; CR, complete remission; FAB, French–American–British; Hb, hemoglobin; LDH, lactate dehydrogenase; PR, partial remission; WBC, white blood cell.

a Number of patients (%).

b Median (range).

c Only 363 patients, including 14 with *TP53* mutation and 349 without, who received conventional intensive induction chemotherapy and then consolidation chemotherapy if CR was achieved, as mentioned in the text, were included in the analysis.

**Table 3:** Association of *TP53* mutation with other gene mutations

*Variables*	*No. of patients with alteration (%)*			P-*value*
	*Whole cohort (*n*=500)*	*TP53-mutated patients (*n*=35)*	*TP53 wild-type patients (*n*=465)*	
*FLT3/*ITD	113 (22.6)	0 (0)	113 (24.3)	0.0002
*FLT3/*TKD	38 (7.6)	0 (0)	38 (8.2)	0.097
*NRAS*	61 (12.2)	2 (5.7)	59 (12.7)	0.2918
*KRAS*	16 (3.2)	0 (0)	16 ((3.4)	0.6175
*PTPN11*	18 (3.6)	2 (5.7)	16 (3.4)	0.3635
*KIT*	15 (3.0)	0 (0)	15 (3.2)	0.6143
*JAK2*	3 (0.6)	0 (0)	3 (0.6)	>0.9999
*WTI*	33 (6.6)	1 (2.9)	32 (6.9)	0.7195
*NPM1*	103 (20.6)	1 (2.9)	102 (21.9)	0.0041
*CEBPA*	66 (13.2)	2 (5.7)	64 (13.8)	0.2957
*RUNX1*	62 (12.4)	2 (5.7)	60 (12.9)	0.2912
*MLL/*PTD	27 (5.4)	0 (0)	27 (5.8)	0.2444
*ASXL1*	50 (10.0)	2 (5.7)	48 (10.3)	0.5613
*IDH1*	27 (5.4)	1 (2.9)	26 (5.6)	0.7115
*IDH2*	55 (11)	1 (2.9)	54 (11.6)	0.1584
*TET2*	66 (13.2)	2 (5.7)	64 (13.8)	0.2957
*DNMT3A*	70 (14.0)	1 (2.9)	69 (14.8)	0.045

**Table 4:** Multivariate analysis (Cox regression) on the disease-free survival and overall survival

*Variables*	*Overall survival*				*Disease-free survival*			
		*95% CI*				*95% CI*		
	*RR*	*Lower*	*Upper*	P	*RR*	*Lower*	*Upper*	P
Age^a^	2.426	1.736	3.391	<0.001^b^	1.431	1.084	1.888	0.011^b^
WBC^c^	2.127	1.481	3.056	<0.001^b^	1.762	1.309	2.370	<0.001^b^
Karyotype^d^	1.971	1.035	3.751	0.039^b^	1.935	1.181	3.168	0.009^b^
*NPM1/FLT3-ITD*^e^	0.304	0.147	0.631	0.001^b^	0.304	0.162	0.567	<0.001^b^
*CEBPA*^f^	0.423	0.211	0.848	0.015^b^	0.596	0.367	0.970	0.037^b^
*IDH2*^g^	0.563	0.292	1.086	0.087	0.937	0.595	1.475	0.778
*WT1*	2.387	1.387	4.109	0.002^b^	2.315	1.505	3.561	<0.001^b^
*RUNX1*	2.103	1.210	3.656	0.008^b^	1.985	1.271	3.100	0.003^b^
*ASXL1*	0.726	0.403	1.306	0.285	0.941	0.544	1.628	0.828
*DNMT3A*	2.204	1.336	3.637	0.002^b^	2.134	1.398	3.256	<0.001^b^
*TP53*	4.684	2.073	10.584	<0.001^b^	2.547	1.244	5.214	0.011^b^

Abbreviations: CI, confidence interval; RR, relative risk.

a Age >50 years relative to age ≤50 years (the reference).

b Statistically significant (*P*<0.05).

c White blood cell (WBC) count >50 000/μl vs ≤50 000/μl.

d Unfavorable cytogenetics vs others.

e *NPM1*^mut^/*FLT3-ITD*^neg^ vs other subtypes.

f *CEBPA*^double-mutation^ vs others.

g *IDH2* mutations included R140 and R172 mutations.

**Table 5:** Sequential studies in the AML patients with *TP53* mutations^a^

*UPN*	*Date*	*Status*	*Karyotype*	*TP53 mutation*	*Other mutations*
10	8/1/2002	Initial	+der(1)t(1;12)(p34;q21),+2,-5,+del(6)(p21p23),+8,+del(9)(p12),-12,-13,add(17)(p13),-19,+20, +21,+mar1,+mar2	R280S	*ASXL1*
	9/23/2002	CR	ND	—	—
11	2003/5/9	Initial	45-46,XY,add(1)(q21),add(6)(q27),-14,-15,add(16)(p12),der(19)add(19)(p13)add(19)(q13),add(22)(q12)[cp8]	L194R	—
	2003/7/10	CR1	NK	L194R	—
	2003/12/18	Relapse 1	del(4)(q2?1),+der(4)t(1;4)(p13;q23),del(6)(q23q27),-10,-14,der(19)add(19)(p13)add(19)(q13),+mar1	L194R	—
12	2003/4/15	Initial	+X,add(1)(p11),-2,dup(3)(p12p13),-5,del(5)(p13p15),der(7)(7pter->7qter::?::12q13->12qter),+8,+10,der(11)dup(11)(q13q25)hsr(11)(q25),-12,+13,-15,-17,+der(?)t(1;?)(p22;?)x2,+der(?)t(?;15)(?;q13),+mar	R248G	—
	2003/7/8	CR1	NK	—	—
	2005/3/1	Relapse 1	ND	R248G	—
	2005/3/25	CR2	NK	—	—
30	2005/1/13	Initial	del(5)(q13q33),-7,+r(16)(p13q24),-17,-17,-18,+der(?)t(?;17)(?;q11),+mar1,+mar2	Y126C	—
	2005/2/14	CR1	NK	—	—
	2005/8/18	Relapse 1	NK	Y126C^b^	—
32	2006/1/4	Initial	NK	V31I	*CEBPA, IDH2*
	2006/5/15	CR	NK	V31I	—
36	1995/5/27	New	del(16)(?q21), +8,t(15;17)(q22;q21)	—	—
	1995/8/10	CR1	NK	ND	—
	2000/10/16	Relapse 1	+8,t(15;17)(q22;q21)	E286K^b^	—
	2000/11/24	CR2	NK	—	—
	2004/3/9	Relapse 2^c^	add(X)(p22),del(1)(q21q44),add(2)(p24),del(3)(p12),-5,add(10)(p13),-16,add(16)(p11),-18,add(18)(q23),add(19)(p13),-22,-22,+mar1,+mar2	E286K	—

Abbreviations: AML, acute myeloid leukemia; CR, complete remission; NK, normal karyotype; ND, not done; UPN, unique patient number.

a The results of serial studies in 125 patients without TP53 mutation at both diagnosis and relapse were not shown in this table.

b No mutation was detected by direct sequencing, but by more sensitive TA cloning technique, TP53 mutation could be found in two of the 45 clones in patient 30 and in one of the 40 clones in patient 36.

c More accurately, this was therapy-related leukemia rather than relapsed leukemia, as the original t(15;17) and other chromosomal changes were no more detected, but complex cytogenetic abnormalities and TP53 mutation emerged instead.
