# Supplementary material for: TP53 mutations in de novo acute myeloid leukemia patients: longitudinal follow-ups show the mutation is stable during disease evolution
Source: Blood Cancer J. 2015 Jul 31;5(7):e331–. doi: 10.1038/bcj.2015.59 (PMC4526785; doi:10.1038/bcj.2015.59)
Supplement: Supplementary Information [file bcj201559x1.doc]

**Supplementary Table 1**

**Primer sequences of *TP53* mutations**

| *TP53*-3F | CCATGGGACTGACTTTCTGC |
| --- | --- |
| *TP53*-4R | GGAGGTGCTTACGCATGTTT |
| *TP53*-5F | GGAGGTGCTTACGCATGTTT |
| *TP53*-6R | TTGCACATCTCATGGGGTTA |
| *TP53*-7F | CCTGCTTGCCACAGGTCT |
| *TP53*-7R | GATGAGAGGTGGATGGGTAGT |
| *TP53*-8F | GGCTTCTCCTCCACCTACCT |
| *TP53*-9R | CGGCATTTTGAGTGTTAGACTG |

**Supplementary Table 2**

**Comparison of immunophenotypes of leukemia cells between AML patients with and without *TP53* mutation**

| **Antigens** | **Total patients examined** | **Percentage of patients with the antigen expression** | | | **P** |
| --- | --- | --- | --- | --- | --- |
|  |  | **Whole cohort** | ***TP53*-Mutated patients** | ***TP53*-wild patients** |  |
| HLA-DR | 476 | 70 | 83.3 | 69.1 | 0.1479 |
| CD13 | 478 | 94.6 | 90 | 94.9 | 0.2183 |
| CD33 | 477 | 91 | 93.3 | 90.8 | >0.9999 |
| CD11b | 149 | 29.8 | 50 | 29 | 0.5817 |
| CD14 | 466 | 13.5 | 3.3 | 14.4 | 0.1023 |
| CD19 | 469 | 7.0 | 3.3 | 7.3 | 0.712 |
| CD10 | 414 | 0.5 | 0 | 0.5 | >0.9999 |
| CD7 | 479 | 20.0 | 23.3 | 19.3 | 0.6335 |
| CD2 | 473 | 4.2 | 3.3 | 4.3 | >0.9999 |
| CD15 | 473 | 44.8 | 36.7 | 45.4 | 0.4488 |
| CD34 | 473 | 65.1 | 82.8 | 63.9 | 0.0443 |
| CD56 | 428 | 26.2 | 40 | 23.5 | 0.5006 |

**Supplementary Table 3**

**Association of *TP53* mutation with chromosomal abnormalities^*^**

| **Variables** | **Total** | ***TP53*-Mutated** | ***TP53*-Wild** | **P value** |
| --- | --- | --- | --- | --- |
| **Karyotype†** |  |  |  |  |
| Favorable | 99 | 1 (1) | 98 (99) | 0.004 |
| Intermediate | 318 | 4 (1.3) | 314 (98.7) | <0.0001 |
| Unfavorable | 65 | 30 (46.2) | 35 (53.8) | <0.0001 |
| Unknown | 18 | 0 (0) | 18 (100) | 0.6323 |
| Normal | 223 | 4 (1.8) | 219 (98.2) | <0.0001 |
| Simple | 208 | 1 (0.5) | 207 (99.5) | <0.0001 |
| Complex | 51 | 30 (58.8) | 21 (41.2) | <0.0001 |
| t(8;21) | 42 | 1 (2.4) | 41 (97.6) | 0.3464 |
| t(15;17) | 38 | 0 (0) | 38 (100) | 0.0976 |
| inv(16) | 19 | 0 (0) | 19 (100) | 0.3844 |
| t(11q23) | 16 | 0 (0) | 16 (100) | 0.6196 |
| t(7;11) | 10 | 0 (0) | 10 (100) | >0.9999 |
| -5/5q-‡ | 2 | 0 (0) | 2 (100) | >0.9999 |
| -7/7q-‡ | 10 | 0 (0) | 10 (100) | >0.9999 |
| +8‡ | 27 | 0 (0) | 27 (100) | 0.2461 |
| +11‡ | 3 | 0 (0) | 3 (100) | >0.9999 |
| +13‡ | 1 | 0 (0) | 1 (100) | >0.9999 |
| +21‡ | 9 | 0 (0) | 9 (100) | >0.9999 |

*Four hundred and eighty-two patients, including 35 *TP53-*mutated and 447 *TP53*-wild patients, had chromosome data at diagnosis.

^†^Favorable, t(15;17), t(8;21), inv (16) ; unfavorable, -7, del(7q), -5, del(5q), 3q abnormality, complex abnormalities; Intermediate, normal karyotype and other abnormalities.

^‡^Only including simple chromosomal abnormalities with 2 or less changes, but not those with complex abnormalities with 3 or more aberrations.
